# Protective Effect of *Artemisia argyi* and Its Flavonoid Constituents against Contrast-Induced Cytotoxicity by Iodixanol in LLC-PK1 Cells

**DOI:** 10.3390/ijms19051387

**Published:** 2018-05-07

**Authors:** Dahae Lee, Chang-Eop Kim, Sa-Yoon Park, Kem Ok Kim, Nguyen Tuan Hiep, Dongho Lee, Hyuk-Jai Jang, Jae Wook Lee, Ki Sung Kang

**Affiliations:** 1 School of Pharmacy, Sungkyunkwan University, Suwon 440-746, Korea; pjsldh@naver.com; 2 College of Korean Medicine, Gachon University, Seongnam 13120, Korea; eopchang@gachon.ac.kr (C.-E.K.); psy9228@gmail.com (S.-Y.P.); 3 Department of Biosystems and Biotechnology, College of Life Science and Biotechnology, Korea University, Seoul 02841, Korea; kikeko520@gmail.com (K.O.K.); nguyentuanhiep.2710@gmail.com (N.T.H.); dongholee@korea.ac.kr (D.L.); 4 Department of Surgery, University of Ulsan College of Medicine, Seoul 05505, Korea; jhj@gnah.co.kr; 5 Natural Constituent Research Center, Korea Institute of Science and Technology, Gangnung 210-340, Korea

**Keywords:** nephrotoxicity, iodixanol, MAPK, caspase, *Artemisia argyi*, flavonoid

## Abstract

Preventive effects and corresponding molecular mechanisms of mugwort (*Artemisia argyi*) extract and its flavonoid constituents on contrast-induced nephrotoxicity were explored in the present study. We treated cultured LLC-PK1 cells with iodixanol to induce contrast-induced nephrotoxicity, and found that *A. argyi* extracts ameliorated the reduction in cellular viability following iodixanol treatment. The anti-apoptotic effect of *A. argyi* extracts on contrast-induced nephrotoxicity was mediated by the inhibition of mitogen-activated protein kinase (MAPK) phosphorylation and the activation of caspases. The flavonoid compounds isolated from *A. argyi* improved the viability of iodixanol-treated cells against contrast-induced nephrotoxicity. Seven compounds (**1**, **2**, **3**, **15**, **16**, **18**, and **19**) from 19 flavonoids exerted a significant protective effect. Based on the in silico oral-bioavailability and drug-likeness assessment, which evaluate the drug potential of these compounds, compound **2** (artemetin) showed the highest oral bioavailability (49.55%) and drug-likeness (0.48) values. We further investigated the compound–target–disease network of compound **2**, and proliferator-activated receptor gamma (PPAR-γ) emerged as a predicted key marker for the treatment of contrast-induced nephrotoxicity. Consequently, compound **2** was the preferred candidate, and its protective effect was mediated by inhibiting the contrast-induced inflammatory response through activation of PPAR-γ and inhibition of MAPK phosphorylation and activation of caspases.

## 1. Introduction

*Artemisia* is a large genus of herbal plants that are commonly used as functional foods and herbal medicine for the treatment of many diseases, including cancer and inflammation, in Korea, China, and Japan [1,2,3]. *Artemisia* extracts and their active compounds show antioxidant and anti-inflammatory activities against stress-related mucosal damage, HCl/EtOH-induced gastric damage, *Helicobacter pylori*-induced gastric damage, microglial neurotoxicity, and pancreatic and hepatic damage [2,4,5,6,7,8,9,10]. The active compounds of *A. asiatica* Nakai have been used in the development of drugs for the treatment of gastritis and Stillen, a standardized extract [2,4].

*Artemisia* plants contain various pharmacologically active ingredients. The results of basic experimental studies into the many health benefits of flavonoids [2,4] have led to an increase in the number of studies on flavonoids from *Artemisia*. Flavonoids exhibit multiple biological effects including antitumor, antioxidant, anti-inflammatory, antiviral, anti-allergic, antithrombotic, and antimutagenic activity, as well as hepatoprotection and renoprotection [11,12,13,14].

Frequently prescribed drugs are one of many factors that damage the kidneys. For example, contrast media facilitate medical image interpretation and are extensively used in cardiac catheterization and angiography and computed tomography [15]. However, the continued use of contrast media is known to increase the number of cases of contrast-induced acute kidney injury [16], which is the third most common cause of hospital-acquired acute kidney injury [15,17]. Although many factors such as vascular disease, hypertension, diabetes, and renal function impairment are involved, the mechanisms of contrast-induced nephrotoxicity are not completely understood. Many in vitro studies have investigated the toxicity of contrast agents using different types of cultured cells, such as renal epithelial cells, mesangial cells, endothelial cells, smooth muscle cells, hepatic cells, human fibroblasts, pulmonary mast cells, human embryonic kidney cells, and human neutrophils. In vitro data have suggested that contrast media induced death of both endothelial and tubular cells in the kidney through cell membrane damage, reactive oxygen species (ROS), inflammation, and activation of the intrinsic apoptotic pathway [15,16,17].

In this study, we investigated whether *A. argyi* extracts and their flavonoid constituents could prevent contrast-induced nephrotoxicity in cultured cells, and explored the underlying molecular mechanisms responsible for these protective effects. The results of this study may aid the identification of the relationship between the structural characteristics of flavonoid compounds and the possible renoprotective effects against contrast-induced nephrotoxicity.

## 2. Results and Discussion

### 2.1. Effect of Artemisia argyi Extract on Iodixanol-Induced Nephrotoxicity in LLC-PK1 Cells

Contrast media is extensively used in X-ray-based imaging techniques for the visualization of internal organs and structures [16,18]. However, in some patients, clinically significant adverse events occur, including permanent impairment of renal function that requires dialysis, longer hospital stays, and unfavorable clinical outcomes [17,18,19,20]. Indeed, contrast-induced nephrotoxicity is a well-known adverse event associated with the use of contrast media; it is the third most common cause of hospital-acquired acute renal failure and accounts for 10–25% of all acute renal failure cases [17,18,19].

**Figure 1:**
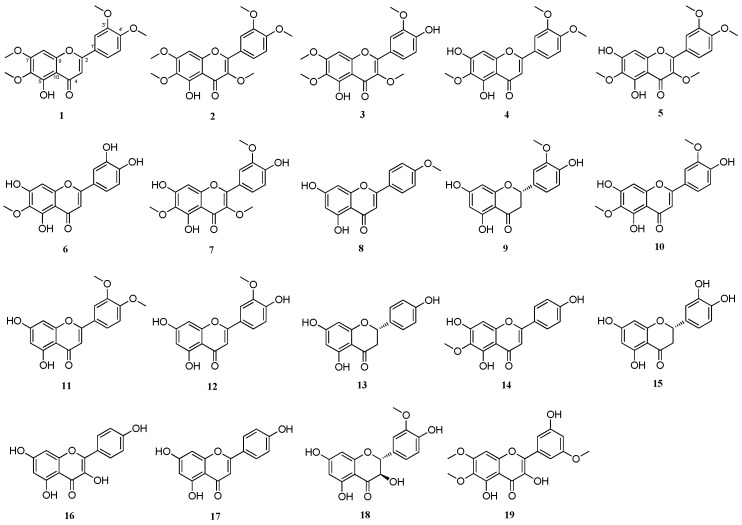
Structures of compounds **1**–**19** from *Artemisia argyi*: 3′-*O*-methyl-eupatorin (**1**); artemetin (**2**); chrysosplenetin (**3**); eupatilin (**4**); bonanzin (**5**); eupafolin (**6**); 3,6,3′-trimethoxy-5,7,4′-trihydroxyflavone (**7**); acacetin (**8**); homoeriodictyol (**9**); jaceosidin (**10**); 3′,4′-dimethoxyluteolin (**11**); 3′-methoxyapigenin (**12**); naringenin (**13**); hispidulin (**14**); 5,7,3′,4-tetrahydroxyflavone (**15**); 3,4′,5,7-tetrahydroxyflavone (**16**); 5,7,4′-trihydroxyflavone (**17**); 2,3-dihydroisorhamnetin (**18**); and apicin (**19**).

**Figure 2:**
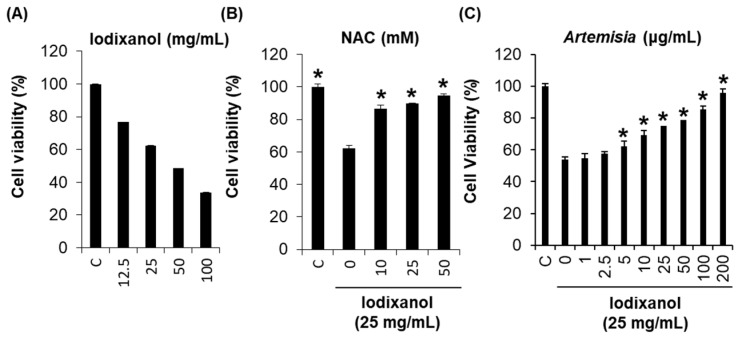
Effect of *Artemisia argyi* extract on iodixanol-induced nephrotoxicity in LLC-PK1 cells: (**A**) cytotoxic effect of iodixanol on viability in LLC-PK1 cells; (**B**) protective effect of *N*-acetyl cysteine (NAC) against iodixanol-induced nephrotoxicity in LLC-PK1 cells; and (**C**) protective effect of *A. argyi* extract against iodixanol-induced nephrotoxicity in LLC-PK1 cells. * *p* < 0.05 compared to the not-treated value.

Based on the possible mechanisms of contrast-induced nephrotoxicity, we assessed the potential protective effect of *A. argyi* extracts and their flavonoid constituents (Figure 1) on contrast-induced cytotoxicity. After treatment with 25 mg/mL iodixanol for 3 h, the LLC-PK1 cell viability reduced by 62.3% (Figure 2A). Several studies have shown that contrast medium can directly induce cytotoxic effects in renal cells. Iodixanol is highly cytotoxic to renal proximal tubular cells, which are a common target of radiographic contrast-induced nephrotoxicity [15,17,18]. In an earlier study about the protective effect of three antioxidants (probucol, ascorbic acid, and *N*-acetyl cysteine (NAC)) on contrast-induced nephrotoxicity, the reduction in cell viability by three contrast media (ioxitalamate, iopromide, and iodixanol) was recovered only by 2 mM NAC co-treatment for 15 min in human embryonic kidney 293T cells [21]. NAC is a potent antioxidant capable of scavenging oxygen-derived free radicals and can prevent oxidative damage [22,23,24]. Recently, several studies have suggested that NAC may prevent contrast-induced nephrotoxicity in both in vitro and in vivo models [17,21,25]. These studies showed that antioxidant pretreatment may reduce the toxicity of radiographic contrast.

In our previous study, we reported that *Artemisia* extracts and the flavonoid constituent eupatilin could prevent cisplatin-induced nephrotoxicity in porcine renal proximal tubular LLC-PK1 cells, and explored the underlying molecular mechanisms of their protective actions [26]. *A. argyi* extracts, which contain eupatilin, may be effective for the treatment of cisplatin-induced nephrotoxicity. Eupatilin is also known to prevent acute ischemia-induced kidney injury in mice through the upregulation of the expression of Hsp70 protein, which has antioxidant and cytoprotective properties against the oxidative stress that is caused by renal tubular cell apoptosis in acute kidney injury [27,28].

Based on these results, we investigated whether *A. argyi* extracts and their flavonoid constituents could prevent contrast-induced nephrotoxicity in porcine renal proximal tubular LLC-PK1 cells. The reduction in cell viability that occurred after treatment with 25 mg/mL iodixanol was recovered by *A. argyi* extract co-treatment in a dose-dependent manner (Figure 2B). In particular, LLC-PK1 cell viability in the group receiving 50 μg/mL *A. argyi* extract co-treatment recovered up to 92.5% of activity compared to that of the iodixanol treatment group. The reduction in cell viability caused by 25 mg/mL iodixanol recovered by up to 94.4% after 50 mM NAC (positive control) co-treatment for 2 h (Figure 2C).

### 2.2. Effect of Artemisia argyi Extract on the Expression of Apoptosis-Related Proteins in LLC-PK1 Cells Exposed to Iodixanol

**Figure 3:**
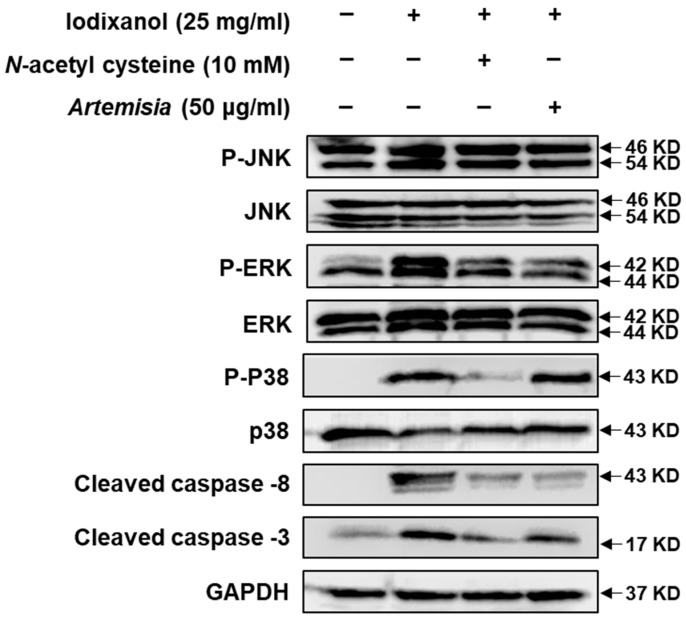
Effects of NAC and *Artemisia argyi* extract on MAPKs (p-JNK, JNK, p-ERK, ERK, p-p38, and p38); caspase-8, -9, and -3 activation; and GAPDH following iodixanol-induced nephrotoxicity on LLC-PK1 cells treated with vehicle control (0.5% DMSO), iodixanol (25 mg/mL), positive control (10 mM NAC), and *A. argyi* (50 μg/mL) for 3 h.

Owing to these observed protective effects, we also investigated the effect of *A. argyi* extract and NAC on MAPKs and caspase-8, -9, and -3 protein expression, which are representative markers of renal damage [25,29], against iodixanol-induced nephrotoxicity in LLC-PK1 cells. Although the clear mechanisms of contrast-induced nephrotoxicity are not well identified, several pathways such as ROS-regulated signaling and apoptotic signaling have been implicated [17,25,30,31,32]. Caspase-1, -3, -8, and -9, Bcl-2, and Bax are known to play a pivotal role in contrast-induced kidney injury as apoptotic signaling pathways [25,29]. In the present study, we observed that the activation of caspase-8 and -3 may contribute to apoptotic renal tubular injury from treatment with contrast agents. However, the activation of caspase-8 and -3 by contrast media was abrogated after co-treatment with the *A. argyi* extract or NAC (Figure 3).

MAP kinases, apoptosis, and inflammatory mediators are known to play important roles in the cellular response to cytokines and external stress signals [33]. In our present study, we observed a marked increase in the phosphorylation of c-Jun-N-terminal kinase (JNK), extracellular-signal-regulated kinase (ERK), and p38 MAP kinases, which are known to be involved in the intracellular signaling associated with cell survival/proliferation in contrast-induced nephrotoxicity. The elevated phosphorylated levels of JNK, ERK, and MAP kinase returned to basal levels within 3 h of treatment with 50 μg/mL *A. argyi* extract. However, in comparison with treatment with 10 mM NAC, there was no change in the p38 MAP kinase levels. Therefore, although *A. argyi* and NAC exert similar antioxidant effects, based on the analysis of three representative MAPKs, their mechanisms of action against contrast-induced nephrotoxicity may differ (Figure 3).

### 2.3. DPPH-Radical-Scavenging Effects of Flavonoid Compounds Isolated from Artemisia argyi Extracts

We attempted to isolate various flavonoids, including eupatilin, from the *A. argyi* extract for the identification of active renoprotective compounds against contrast-induced nephrotoxicity. Nineteen previously reported flavonoids were isolated and identified by the comparison of their spectroscopic data with published literature data (see Supplementary Materials). For the flavonoids isolated from *A*. *argyi*, the free radical-scavenging activity against DPPH was determined spectrophotometrically, and we reported the IC_50_ value, which is the sample concentration at which 50% of the DPPH radicals were scavenged (Table 1). Five compounds (**6**, **15**, **16**, **18**, and **19**) from nineteen flavonoids in the *A. argyi* extract showed free radical-scavenging activity. Among them, the effects of compound **6** (eupafolin) and compound **15** (5,7,3′,4′-tetrahydroxyflavone) revealed significant donation of electrons to the stable free radical DPPH at a similar level to ascorbic acid (positive control). The excellent antioxidant, anti-inflammatory, and antiproliferative effects of these compounds have been previously reported [34,35,36].

### 2.4. Comparison of the Protective Effects of the Flavonoid Compounds Isolated from Artemisia argyi Extracts against Iodixanol-Induced Nephrotoxicity in LLC-PK1 Cells

**Figure 4:**
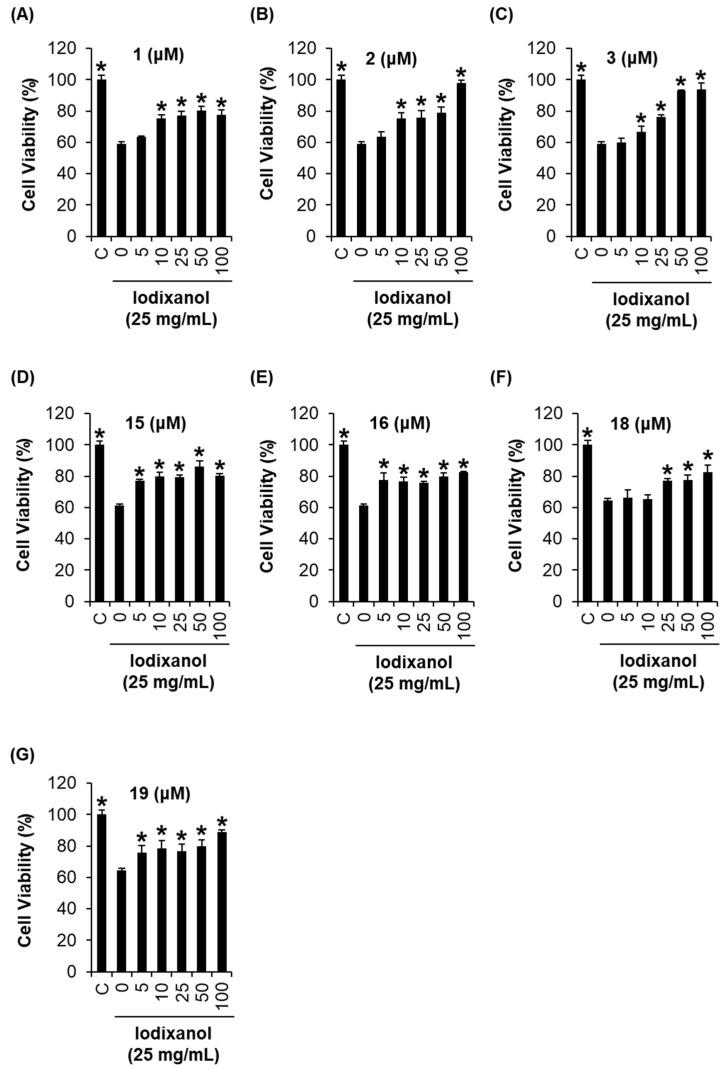
Comparison of the protective effects of the flavonoid compounds isolated from *Artemisia argyi* extracts against iodixanol-induced nephrotoxicity in LLC-PK1 cells: (**A**) protective effect of 3′-*O*-methyl-eupatorin (**1**); (**B**) protective effect of artemetin (**2**); (**C**) protective effect of chrysosplenetin (**3**); (**D**) protective effect of 5,7,3′,4-tetrahydroxyflavone (**15**); (**E**) protective effect of 3,4′,5,7-tetrahydroxyflavone (**16**); (**F**) protective effect of 2,3-dihydroisorhamnetin (**18**); (**G**) protective effect of apicin (**19**). * *p* < 0.05 compared to the not-treated value.

To investigate the relationship between the structural characteristics of flavonoids and the renoprotective effect on contrast-induced nephrotoxicity in porcine renal proximal tubular LLC-PK1 cells, we treated the cells with various concentrations of flavonoids (Figure 4A–G). Seven compounds (**1**, **2**, **3**, **15**, **16**, **18**, and **19**) from nineteen flavonoids exerted a protective effect. The reduction in cell viability by iodixanol was recovered by more than 80% by co-treatment with all seven compounds at 100 μM. These results suggest that the antioxidant activity may also partly contribute to the protective effect on contrast-induced nephrotoxicity.

### 2.5. DL and OB Evaluation of Flavonoid Compounds with Profound Protective Effects against Iodixanol-Induced Nephrotoxicity in LLC-PK1 Cells

We further investigated the drug potential of the four most efficacious compounds (**2**, **3**, **18**, and **19**) to propose the most reliable candidates for the treatment of contrast-induced nephrotoxicity. The properties of oral-bioavailability (OB) and drug-likeness (DL), which are predicted values using in silico models, were evaluated, and all the four compounds showed feasible properties given the threshold values of OB ≥ 30% and DL ≥ 0.18 (Table 2). In particular, compound **2** had the highest OB (49.55%) and DL values (0.48), which made it the preferred candidate.

### 2.6. Compound-Target Network of Compound **2**

**Figure 5:**
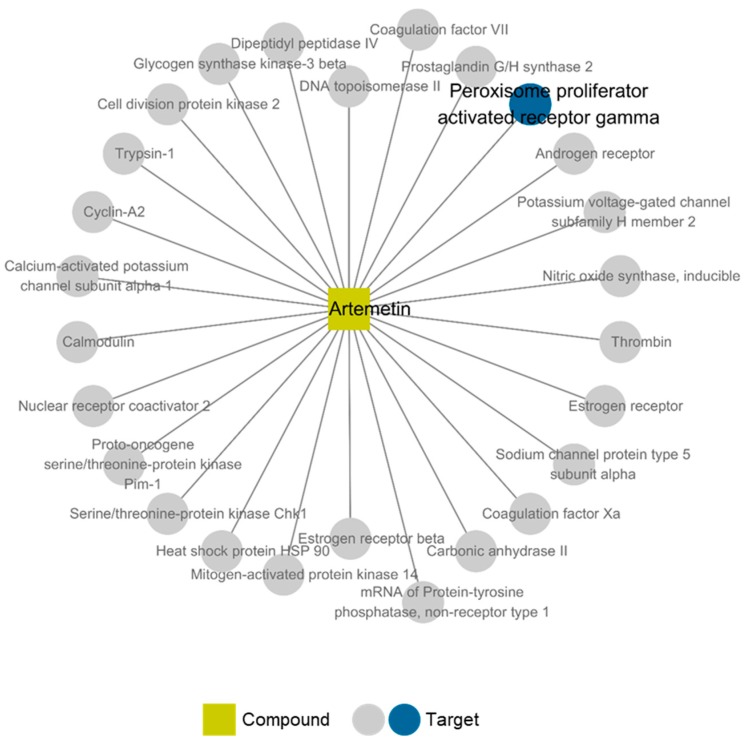
Compound-target network of compound **2**. The network is constructed with one compound and 26 corresponding targets. Disease nodes were omitted for clear visualization.

Furthermore, we conducted in silico network pharmacological analysis to investigate the possible mechanism of compound **2** on nephrotoxicity. The compound–target–disease network of compound **2** was constructed using TCMSP (see Materials and Methods). We found that 26 targets were linked with compound **2** in the constructed network. Among them, we focused on peroxisome proliferator-activated receptor gamma (PPAR-γ), which was related to the disease node “inflammation” (Figure 5). The nuclear receptor PPAR-γ regulates transcription factors that involved in lipid metabolism, fatty acid metabolism, glucose homeostasis, cell proliferation, inflammation, and related metabolic disorders [37,38,39]. In the kidney, various renal cell types including the proximal tubules and medullary collecting duct cells have endogenous PPAR-γ expression and activity. Previous studies have shown that the suppression of PPAR-γ involved in p53 and Bax interaction in renal tubular cell apoptosis [40] resulted in renal injury associated with ischemia/reperfusion in rats. In addition, the later suppression of PPAR-γ may result in hyperuricemia patients with chronic renal injury [41]. Therefore, expression of PPAR-γ in the kidney may play an important role in maintaining normal renal function [42].

### 2.7. Effects of Artemetin on the Morphological Changes, Degree of Nuclear Condensation and Protein Expression of PPAR-γ in the Iodixanol-Induced Nephrotoxicity of LLC-PK1 Cells

**Figure 6:**
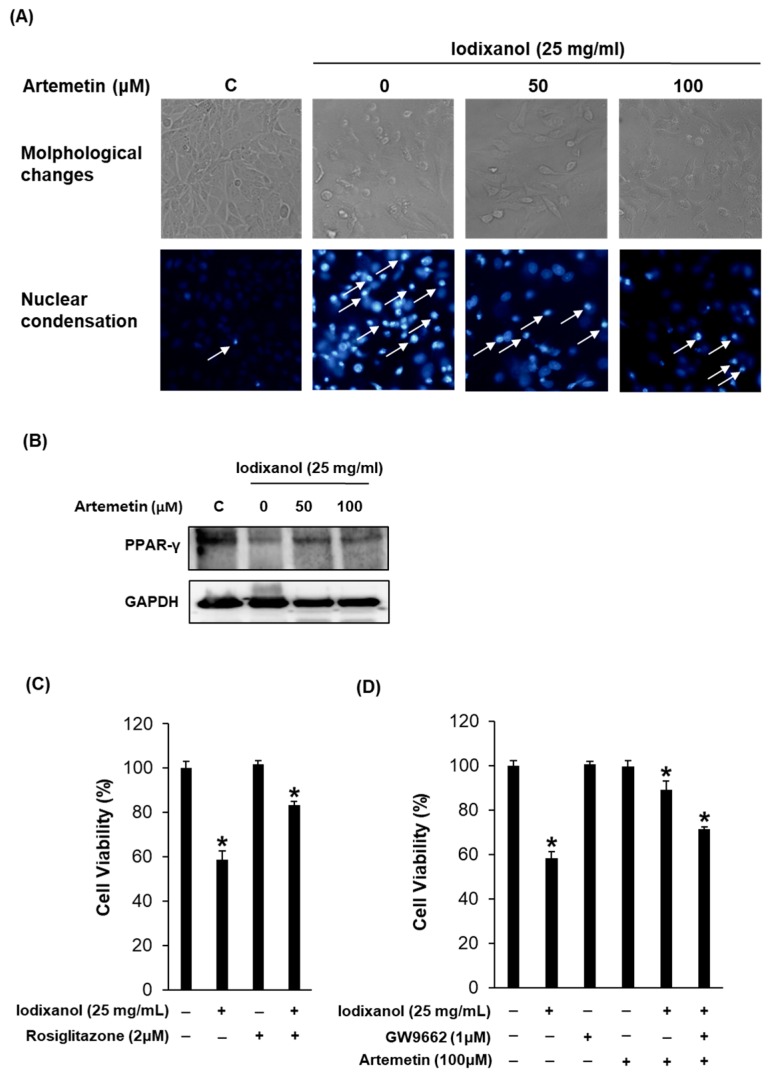
Effects of artemetin on the morphological changes, degree of nuclear condensation and protein expression of PPAR-γ in the iodixanol-induced nephrotoxicity of LLC-PK1 cells treated with vehicle control (0.5% DMSO), iodixanol (25 mg/mL), and artemetin (50 and 100 μM) for 3 h: (**A**) morphological changes and degree of nuclear condensation as determined by measuring the level of Hoechst 33342 fluorescence; (**B**) protein expression of PPAR-γ; (**C**) effect of PPAR-γ ligand (rosiglitazone) on iodixanol-induced cytotoxicity in LLC-PK1 cells; and (**D**) effect of PPAR-γ antagonist (GW9662) on iodixanol-induced cytotoxicity in LLC-PK1 cells. * *p* < 0.05 compared to the not-treated value.

Consequently, we focused on the identification of the protective mechanism of compound **2** on contrast-induced LLC-PK1 cell damage including PPAR-γ as a predicted key marker of the compound–target–disease network of compound **2**. As shown in Figure 6A, exposure to contrast media induced morphological changes in LLC-PK1 cells, which appeared blebbed and shrunk, as detected by phase-contrast inverted microscopy. These abnormal morphological changes were recovered in cells exposed to 50 and 100 μM compound **2** (Figure 6A). In addition, the nuclear condensation induced by iodixanol, as detected spectrofluorometrically, was significantly ameliorated after treatments of cells with compound **2** at concentrations of 50 and 100 μM (Figure 6A). In addition, we observed that the suppression of PPAR-γ may contribute to apoptotic renal tubular injury from treatment with contrast agents. However, this suppression of PPAR-γ by contrast media returned to basal levels upon treatment of cells with compound **2** (Figure 6B).

Based on the possible mechanisms of PPAR-γ in the iodixanol-induced cytotoxicity of LLC-PK1 cells, we assessed the protective effect of PPAR-γ agonist rosiglitazone (Figure 6C) on iodixanol-induced cytotoxicity. After treatment with 25 mg/mL iodixanol, the LLC-PK1 cell viability was reduced to 58.7% (Figure 6C). However, this reduced cell viability by iodixanol was recovered to 83.4% by co-treatment with 1 μM rosiglitazone. In addition, we assessed the effect of PPAR-γ selective antagonist GW9662 on the protection effect of artemetin on iodixanol-induced cytotoxicity. GW9662 and artemetin used at concentrations that had no considerable effect on cell viability. The reduction in cell viability by iodixanol was recovered by 89.0% by co-treatment with 100 μM artemetin, and this protective effect was reduced by 71.3% after co-treatment with 1 μM GW9662 (Figure 6D). Taken together, our data suggest that artemetin exerts its protection effect through PPAR-γ in LLC-PK1 cells.

### 2.8. Effects of Artemetin on Iodixanol-Induced Apoptosis in LLC-PK1 Cells

**Figure 7:**
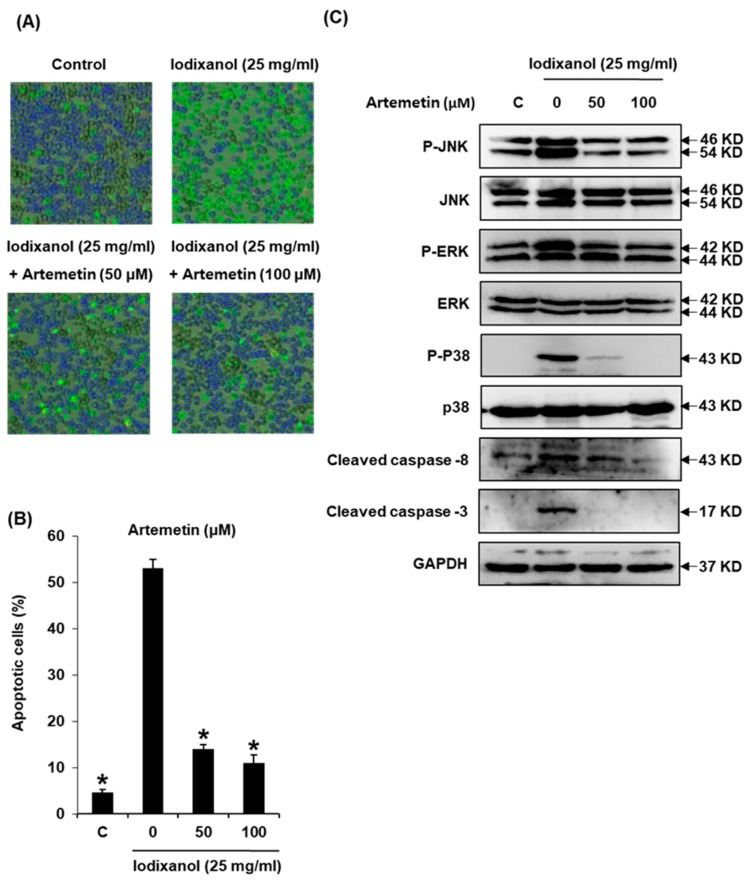
Effects of artemetin on apoptosis in LLC-PK1 cells exposed to iodixanol (25 mg/mL) for 24 h by image-based cytometric assay and western blot analysis: (**A**) representative images for apoptosis detection; (**B**) percentage of Annexin V-positive-stained apoptotic cells; and (**C**) protein expression of Bcl-2, Bax, cleaved caspase-8, cleaved caspase-3 and JNK. * *p* < 0.05 compared to the not-treated value.

We further explored whether compound **2** could decrease apoptosis in LLC-PK1 cells exposed to contrast by using the annexin V Alexa Fluor 488 and propidium iodide staining (Figure 7A). Apoptotic cell death, observed via staining with annexin V, increased from 4.6 ± 0.5% to 53.0 ± 2.0% after 25 mg/mL contrast treatment, whereas a decrease by 14.0 ± 1.0% and 11.0 ± 1.7% was observed after treatment with 50 and 100 μM of compound **2**, respectively, as shown in the quantified graph for the percentage of apoptotic cells (Figure 7B). Based on the results of protein analysis associated with inflammation and apoptosis via Western blot, the increased phosphorylation of JNK and ERK MAPKs by iodixanol returned to basal levels with treatment of compound **2**. Furthermore, the activation of caspase-8 and -3 by iodixanol was abrogated after treatment with the compound **2** (Figure 7C). Therefore, the protective effect of compound **2** on iodixanol-induced apoptosis in LLC-PK1 cells was mediated by the inhibition of MAPK phosphorylation and caspase activation.

## 3. Materials and Methods

### 3.1. Chemicals and Reagents

Iodixanol, rosiglitazone and GW9662 were purchased from Sigma Aldrich (St. Louis, MO, USA). Ez-Cytox cell viability assay kit was purchased form Dail Lab Service Co. (Seoul, Korea). Dulbecco’s Modified Eagle’s Medium (DMEM) was purchased from Cellgro (Manassas, VA, USA). FBS was purchased from Invitrogen Co. (Grand Island, NY, USA). Pierce™ BCA Protein Assay Kit was purchased from Thermo Scientific (Waltham, MA, USA). ECL Advance Western Blotting Detection Reagent was purchased from GE Healthcare (Amersham, UK). RIPA buffer, antibodies for p38 MAP kinase, phospho-p38, p44/42 MAP kinase (Erk1/2), phospho-p44/42 (Erk1/2), JNK, phospho-JNK, cleaved caspase-8, cleaved caspase-3, PPAR-γ and glyceraldehyde 3-phosphate dehydrogenase (GAPDH), and horseradish peroxidase (HRP) conjugated anti-rabbit antibodies were purchased from Cell Signaling (Boston, MA, USA).

### 3.2. Plant Materials

Leaves of *A. argyi* H.Lév. & Vaniot were purchased in April 2013 from Gyeongdong herbal medicine market (Seoul, South Korea), and identified by Je-Hyun Lee from the College of Oriental Medicine, Dongguk University, Gyeongju, Korea. A voucher specimen (accession number: AA1-103-130429) was deposited at the Department of Biosystems and Biotechnology, Korea University, Seoul, Korea.

### 3.3. Extraction and Isolation of Flavonoids from A. argyi

The dried leaves of *A. argyi* (3 kg) were subjected to extraction three times with MeOH (18 L, 9 L, 9 L) at room temperature. After removal of the organic solvent in vacuo, the MeOH extract (420 g) was dissolved in water (3.9 L). The aqueous solution was sequentially partitioned with *n*-hexane (3 × 1.3 L) and EtOAc (3 × 1.3 L) to yield the dried soluble extracts of *n*-hexane (KO1-103-1, 50 g) and EtOAc (KO1-103-2, 78 g). The EtOAc extract (70 g) was subjected to silica gel column chromatography (CC) and eluted with a gradient of CHCl_3_-MeOH (1:0 to 1:1) to yield nine fractions (KO1-107-1–KO1-107-9). Fraction KO1-107-5 (25 g) was separated again via silica gel CC and eluted with a gradient of CHCl_3_-acetone (1:0 to 1:1) to yield eight fractions (KO1-120-1–KO1-120-8). The compound eupatilin (**4**, 559.8 mg) [43,44] was precipitated from fraction KO1-120-3, and the remainder of this fraction (4.14 g) was subjected to Sephadex LH-20 CC and eluted with CHCl_3_-MeOH (1:1) to yield 11 fractions (KO1-122-1–KO1-122-11). KO1-122-5 (1.18 g) was fractionated via MPLC silica gel CC and eluted with CHCl_3_-MeOH (97:3) to yield the fractions KO1-123-1–KO1-123-8. The fractions KO1-123-1–KO1-123-3 were combined (375 mg) and fractionated using MPLC silica gel CC and eluted with a gradient of *n*-hexane-EtOAc (1:0 to 1:1) to yield seven fractions (KO1-126-1–KO1-126-7). Fraction KO1-126-6 (30.5 mg) was purified via preparative HPLC (70% MeOH in H_2_O) to yield artemetin (**2**, 5.0 mg) [45]. Additionally, 3′-*O*-methyl-eupatorin (**1**, 3.2 mg) [46] was precipitated from fraction KO1-126-7 (16.0 mg). Fraction KO1-123-4 (26 mg) was purified by preparative HPLC (60-100% MeOH in H_2_O) to yield the compound bonanzin (**5**, 2.5 mg) [47]. KO1-122-6 (570 mg) was subjected to silica gel CC and eluted with a gradient mixture of *n*-hexane-EtOAc (1:0 to 0:1), which resulted in the preparation of fractions TH5-67-1–TH5-67-5. Fraction TH5-67-5 (49.2 mg) was separated by preparative HPLC (65% MeOH in H_2_O) to yield chrysosplenetin (**3**, 7.5 mg) [48]. Fraction KO1-122-8 (86 mg) was separated by preparative HPLC (50–75% MeOH in H_2_O) to yield 3,6,3′-trimethoxy-5,7,4′-trihydroxyflavone (**7**, 12.0 mg) [49]. Fraction KO1-122-10 (40.5 mg) was separated by preparative HPLC (35–100% MeOH in H_2_O) to yield acacetin (**8**, 2.2 mg) [50]. KO1-120-4 (5.26 g) was subjected to Sephadex LH-20 CC and eluted with CHCl_3_-MeOH (1:1) to yield eight fractions (KO1-124-1–KO1-124-8). The compound jaceosidin (**10**, 339 mg) [49] was precipitated from fraction KO1-124-7. Fraction KO1-124-8 (186 mg) was separated by preparative HPLC (35–75% MeOH in H_2_O) to yield homoeriodictyol (**9**, 10.1 mg) [51] and 3′,4′-dimethoxyluteolin (**11**, 3.3 mg) [52]. Fraction KO1-107-6 (6.1 g) was separated using Sephadex LH-20 CC and eluted with CHCl_3_-MeOH (1:1) to yield seven fractions (TH5-47-1–TH5-47-7). Fraction TH5-47-4 (314.2 mg) was subjected to silica gel CC with a gradient mixture of *n*-hexane-EtOAc (0:1 to 1:0) yielding fractions TH5-51-1–TH5-51-10. The purification of fraction TH5-51-8 (11.0 mg) was carried out by silica gel CC and eluted with a mixture of CHCl_3_-MeOH (99:1), which led to the isolation of apicin (**19**, 2.8 mg) [53]. Fraction TH5-51-5 (61.0 mg) was purified by preparative HPLC (40–70% MeOH in H_2_O) to yield crystallized 3′-methoxyapigenin (**12**, 2.7 mg) [54]. Fraction TH5-51-6 (125.9 mg) was separated by preparative HPLC (40–70% MeOH in H_2_O) to yield naringenin (**13**, 20.6 mg) [55], hispidulin (**14**, 12.0 mg) [56], and 2,3-dihydroisorhamnetin (**18**, 6.1 mg) [57]. Fraction KO1-107-7 (7.1 g) was separated using Sephadex LH-20 CC and eluted with CHCl_3_-MeOH (2:1) to yield five fractions (TH5-57-1–TH5-57-5). Fraction TH5-57-4 (377.8 mg) was subjected to silica gel CC with a gradient mixture of n-hexane-EtOAc (0:1 to 1:0), which resulted in fractions TH5-61-1–TH5-61-7. Fraction TH5-61-3 (92.3 mg) was purified by preparative HPLC (50–75% MeOH in H_2_O) to obtain eupafolin (**6**, 36.5 mg) [58] and 5,7,4′-trihydroxyflavone (**17**, 9.8 mg) [59]. Fraction TH5-57-5 (50.9 mg) was purified by preparative HPLC (25-85% MeOH in H_2_O) to obtain 5,7,3′,4′-tetrahydroxyflavone (**15**, 31.2 mg) [60] and 3,4′,5,7-tetrahydroxyflavone (**16**, 3.2 mg) [61].

### 3.4. General Experimental Procedures

NMR spectra were recorded using a Varian 500-MHz NMR spectrometer (Inova 500 Spectrometer, Varian, Palo Alto, CA, USA) with tetramethylsilane as an internal standard, and chemical shifts were recorded in ppm (δ). ESI-MS was conducted using an LCQ Fleet Ion Trap mass spectrometer (Thermo Scientific, Madison, WI, USA). Column chromatography (CC) was performed using silica gel (Kieselgel 60, 230–400 mesh; Merck) and Sephadex LH-20 (18–111 μm; GE Healthcare AB, Stockholm, Sweden). Thin-layer chromatography was performed using pre-coated silica Q-gel 60 F254 plates (0.25 mm; Merck), and preparative HPLC was carried out using the Varian Prostar 210 system with a YMC-Pack ODS-A column (5 μm, 250 × 20 mm i.d.; YMC, Kyoto, Japan). MPLC (Isolera^TM^ One) silica gel column chromatography was performed on Biotage^®^ equipment with a Biotage^®^ SNAP cartridge HP-SIL (25–100 g) silica gel column.

### 3.5. DPPH Radical-Scavenging Assay

The DPPH radical-scavenging assay analyzes antioxidant behavior based on an electron-transfer reaction. The change in color from purple to yellow is observed as DPPH reacts with any antioxidant compounds present. The reaction mixture consisted of a solution of antioxidant compounds (100 μL) and an equal volume of DPPH solution (0.1 mM) in ethanol, and was incubated for 30 min at room temperature in the dark. Ascorbic acid was used as the positive control. DPPH reaction was monitored by measuring the absorbance (Abs.) at 517 nm by using a microplate reader (PowerWave XS; Bio-Tek Instruments, Winooski, VT, USA). The radical-scavenging activity is presented as an IC_50_ value, which is the concentration of the antioxidant compounds required to inhibit 50% of the free radicals, and was calculated using the following equation: Scavenging activity (%) = [(Control Abs. − Sample Abs.)/Control Abs.] × 100.

### 3.6. Cell Culture

LLC-PK1 (pig kidney epithelium, CL-101) cells were purchased from American Type Culture Collection (Rockville, MD, USA), and were cultured in DMEM (Cellgro, Manassas, VA, USA) supplemented with 10% FBS, 1% penicillin/streptomycin (Invitrogen Co., Grand Island, NY, USA), and 4 mM l-glutamine in an atmosphere of 5% CO_2_ at 37 °C. The cells were routinely passaged before they reached 80% confluence, and the culture medium was replaced with fresh medium every 2 days.

### 3.7. Renoprotective Effect against Iodixanol-Induced Damage in Kidney Cells

The experiment was performed according to the method reported previously in our study about renoprotective effects against iodixanol-induced damage in kidney cells [62]. Briefly, the cells were seeded onto 96-well culture plates at 1 × 10^4^ cells/well and allowed to adhere for 24 h. Cells were treated with the vehicle control (0.5% DMSO), positive control (10 mM NAC), indicated concentrations of *A. argyi* extract, and its flavonoid constituents. After incubation for 2 h, 25 mg/mL iodixanol was added to each well and incubated for a further 3 h. Cell viability was determined using the Ez-Cytox cell viability detection kit in accordance with the manufacturer’s instructions, and evaluated by measuring the absorbance at 450 nm using a microplate reader (PowerWave XS; Bio-Tek Instruments, Winooski, VT, USA).

### 3.8. Effect of PPAR-γ Ligand (Rosiglitazone) and PPAR-γ Antagonist (GW9662) against Iodixanol-Induced Damage in Kidney Cells

To identify the effect of rosiglitazone on iodixanol-induced cytotoxicity, the cells were seeded onto 96-well culture plates at 1 × 10^4^ cells/well and allowed to adhere for 24 h. Cells were treated with the vehicle control (0.5% DMSO), iodixanol (25 mg/mL) or rosiglitazone (1 μM) alone and also treated with combinations of rosiglitazone (1 μM) and iodixanol (25 mg/mL). To verify the effect of GW9662 in the presence of the artemetin, the cells were seeded onto 96-well culture plates at 1 × 10^4^ cells/well and allowed to adhere for 24 h. Cells were treated with the vehicle control (0.5% DMSO), iodixanol (25 mg/mL), GW9662 (1 μM) or artemetin (100 μM) alone and also treated with combinations of iodixanol (25 mg/mL) and artemetin (100 μM) or combinations of iodixanol (25 mg/mL), artemetin (100 μM) and GW9662 (1 μM). Cell viability was determined using the Ez-Cytox cell viability detection kit in accordance with the manufacturer’s instructions, and evaluated by measuring the absorbance at 450 nm using a microplate reader.

### 3.9. Nuclear Staining with Hoechst 33342

Cells were seeded onto 6-well culture plates at 4 × 10^5^ cells/well. allowed to adhere for 24 h, and were treated with the vehicle control (0.5% DMSO) and compound **2**. After incubation for 2 h, 25 mg/mL iodixanol was added to each well. After incubation for 3 h, Hoechst 33342 solution was added to each well and incubated for a further 10 min. Stained cells were visualized by fluorescence microscopy.

### 3.10. Image-Based Cytometric Assay

Cells were seeded onto 6-well culture plates at 4 × 10^5^ cells/well and allowed to adhere for 24 h. Cells were treated with the vehicle control (0.5% DMSO) and compound **2**. After incubation for 2 h, annexin V Alexa Fluor 488 and propidium iodide were added to each well and incubated for a further 30 min in darkness. Apoptotic cells (stained green with annexin V Alexa Fluor 488), dead cells (stained red with propidium iodide and green with annexin V Alexa Fluor 488), and live cells (unstained) were visualized and counted by using a Tali image-based cytometer (Invitrogen, CA, USA) [63].

### 3.11. Western Blotting Analysis

LLC-PK1 cells were seeded onto 6-well plates at 4 × 10^5^ cells/well and treated with the vehicle control (0.5% DMSO), *A. argyi* extract (50 μg/mL), compound **2**, and NAC (10 mM) as the positive control. After incubation for 2 h, 25 mg/mL iodixanol was added to each well and incubated for 3 h. The cells were lysed with RIPA buffer and supplemented with 1 mM phenylmethylsulfonyl fluoride (PMSF) immediately before use. Protein concentration was determined using the Pierce™ BCA Protein Assay Kit according to the manufacturer’s instructions, and bovine serum albumin (BSA) was used as the standard protein. Equal amounts (20 μg/lane) of protein sample were separated via electrophoresis in 10% sodium dodecyl sulfate-polyacrylamide gel and transferred onto PVDF transfer membranes [64]. Proteins were analyzed with epitope-specific primary antibodies to JNK, phospho-JNK, p44/42 MAP Kinase (ERK), phospho-p44/42 (pERK), p38 MAP Kinase, phospho-p38, cleaved caspase-8, cleaved caspase-3, PPAR-γ, glyceraldehyde 3-phosphate dehydrogenase (GAPDH), and horseradish peroxidase (HRP) conjugated anti-rabbit secondary antibodies. Bound antibodies were detected using ECL Advance Western Blotting Detection Reagents and visualized on a FUSION Solo Chemiluminescence System (PEQLAB Biotechnologie GmbH, Erlangen, Germany).

### 3.12. Oral-Bioavailability (OB) and Drug-Likeness (DL) Evaluation

OB and DL values were obtained from the Traditional Chinese Medicine Systems Pharmacology Database (TCMSP, http://tcmspnw.com). OB is one of the most important pharmacokinetic properties for drug discovery. In TCMSP, OB values are calculated using in silico screening model OBioavail 1.1 [65]. The model was constructed using 805 structurally diverse drugs and drug-like molecules, and has been proven as an effective drug-screening model in many previous studies.

The property of drug-likeness is a qualitative concept used in drug design. The DLs determined in TCMSP are estimated values using molecular descriptors based on Lipinski’s rule of five [66] and Tanimoto coefficients [67], which measure the structural similarities between herbal ingredients and drugs in the Drugbank database (http://www.drugbank.ca/). In this study, the threshold values for evaluation were OB ≥ 30% and DL ≥ 0.18, which are the default suggested thresholds for TCMSP.

### 3.13. Network Analysis

Compound–target–disease network is a tripartite network with three types of nodes: compounds, targets, and diseases. The edges between compounds and targets are defined as compound–target interactions (1 or 0). To construct a network, compound–target interaction information was extracted from TCMSP for all pairs of candidate compounds and target proteins in the database. This included experimentally validated interactions, but most of the interactions were predicted interactions, based on the machine learning methods (support vector machine and random forest) with validated drug–target interaction datasets. The performance of these predictive methods for compound–target interactions have been proven to be reliable [46]. The information to define edges between targets and diseases was extracted from PharmGKB (http://www.pharmgkb.org) and Therapeutic Targets Database (http://bidd.nus.edu.sg/BIDD-Databases/TTD/TTD.asp).

### 3.14. Statistical Analysis

Statistical significance was determined by analysis of variance (ANOVA) followed by a multiple comparison test with a Bonferroni adjustment. A value of *p* < 0.05 was considered statistically significant. The analysis was performed using SPSS ver. 19.0 (SPSS Inc., Chicago, IL, USA).

## 4. Conclusions

*A. argyi* extracts mitigated the reduced viability of iodixanol-treated LLC-PK1 kidney cells. The anti-apoptotic effects of *A. argyi* extracts on contrast-induced nephrotoxicity was mediated by the suppression of MAPKs and activation of caspases. In addition, flavonoid compounds isolated from the *A. argyi* extract improved the reduced viability of the cells treated with iodixanol. Compound **2** isolated from the *A. argyi* extract exhibited anti-apoptotic effects mediated by the suppression of MAPKs and activation of caspases. Moreover, PPAR-γ levels following suppression by contrast media returned to basal levels after treatment with compound **2**. Compound **2** (artemetin) was the preferred candidate and its protective effect was mediated by inhibition of contrast-induced inflammatory response by activating PPAR-γ and inhibiting MAPK phosphorylation and the activation of caspases. Further studies on the in vivo effects to confirm the use of *A. argyi* extracts and their flavonoid constituents are necessary to prove their beneficial effects in reducing the adverse effects of contrast agents in the kidney.

## Figures and Tables

**Table 1:** DPPH-radical-scavenging effects of flavonoid compounds isolated from *Artemisia argyi* extracts.

Compound	IC_50_ (μM)
**6**	5.04 ± 2.11
**15**	8.21 ± 1.74
**16**	37.06 ± 1.41
**18**	39.58 ± 1.13
**19**	19.05 ± 2.39
Ascorbic acid	2.06 ± 3.12

**Table 2:** DL and OB evaluation of flavonoid compounds with profound protective effects against iodixanol-induced nephrotoxicity in cells. MW, molecular weight; AlogP, octanol–water partition coefficient; Hdon, number of hydrogen-bond donors; Hacc, number of hydrogen-bond acceptors.

Compound	OB (%)	DL	MW	AlogP	Hdon	Hacc
**2**	49.55	0.48	388.4	2.31	1	8
**3**	27.36	0.44	374.37	2.05	2	8
**18**	36.16	0.25	286.25	2.07	4	6
**19**	41.88	0.24	286.25	1.77	4	6

## Supplementary Materials

The following are available online at http://www.mdpi.com/1422-0067/19/5/1387/s1.

## References

[1] Hajdu Z., Hohmann J., Forgo P., Mathe I., Molnar J., Zupko I. (2014). Antiproliferative activity of artemisia asiatica extract and its constituents on human tumor cell lines. Planta Med..

[2] Jeong D., Yi Y.S., Sung G.H., Yang W.S., Park J.G., Yoon K., Yoon D.H., Song C., Lee Y., Rhee M.H. (2014). Anti-inflammatory activities and mechanisms of artemisia asiatica ethanol extract. J. Ethnopharmacol..

[3] Molnar J., Szebeni G.J., Csupor-Loffler B., Hajdu Z., Szekeres T., Saiko P., Ocsovszki I., Puskas L.G., Hohmann J., Zupko I. (2016). Investigation of the antiproliferative properties of natural sesquiterpenes from artemisia asiatica and onopordum acanthium on HL-60 cells in vitro. Int. J. Mol. Sci..

[4] Park J.M., Han Y.M., Lee J.S., Ko K.H., Hong S.P., Kim E.H., Hahm K.B. (2015). Nrf2-mediated mucoprotective and anti-inflammatory actions of artemisia extracts led to attenuate stress related mucosal damages. J. Clin. Biochem. Nutr..

[5] Park S.W., Oh T.Y., Kim Y.S., Sim H., Park S.J., Jang E.J., Park J.S., Baik H.W., Hahm K.B. (2008). Artemisia asiatica extracts protect against ethanol-induced injury in gastric mucosa of rats. J. Gastroenterol. Hepatol..

[6] Oh T.Y., Ahn G.J., Choi S.M., Ahn B.O., Kim W.B. (2005). Increased susceptibility of ethanol-treated gastric mucosa to naproxen and its inhibition by DA-9601, an Artemisia asiatica extract. World J. Gastroenterol..

[7] Lim B.O., Chung H.G., Lee W.H., Lee H.W., Suk K. (2008). Inhibition of microglial neurotoxicity by ethanol extract of Artemisia asiatica Nakai. Phytother. Res..

[8] Choi S.C., Choi E.J., Oh H.M., Lee S., Lee J.K., Lee M.S., Shin Y.I., Choi S.J., Chae J.R., Lee K.M. (2006). DA-9601, a standardized extract of Artemisia asiatica, blocks TNF-alpha-induced IL-8 and CCL20 production by inhibiting p38 kinase and NF-kappa B pathways in human gastric epithelial cells. World J. Gastroenterol..

[9] Hahm K.B., Kim J.H., You B.M., Kim Y.S., Cho S.W., Yim H., Ahn B.O., Kim W.B. (1998). Induction of apoptosis with an extract of Artemisia asiatica attenuates the severity of cerulein-induced pancreatitis in rats. Pancreas.

[10] Chang J.W., Hwang H.S., Kim Y.S., Kim H.J., Shin Y.S., Jittreetat T., Kim C.H. (2015). Protective effect of Artemisia asiatica (Pamp.) Nakai ex Kitam ethanol extract against cisplatin-induced apoptosis of human HaCaT keratinocytes: Involvement of NF-kappa B- and Bcl-2-controlled mitochondrial signaling. Phytomedicine.

[11] Li Y.Y., Wu H., Dong Y.G., Lin B.O., Xu G., Ma Y.B. (2015). Application of eupatilin in the treatment of osteosarcoma. Oncol. Lett..

[12] Nijveldt R.J., van Nood E., van Hoorn D.E., Boelens P.G., van Norren K., van Leeuwen P.A. (2001). Flavonoids: A review of probable mechanisms of action and potential applications. Am. J. Clin. Nutr..

[13] Ahlenstiel T., Burkhardt G., Kohler H., Kuhlmann M.K. (2003). Bioflavonoids attenuate renal proximal tubular cell injury during cold preservation in Euro-Collins and University of Wisconsin solutions. Kidney Int..

[14] Bosetti C., Rossi M., McLaughlin J.K., Negri E., Talamini R., Lagiou P., Montella M., Ramazzotti V., Franceschi S., LaVecchia C. (2007). Flavonoids and the risk of renal cell carcinoma. Cancer Epidemiol. Biomark. Prev..

[15] Sadat U. (2013). Radiographic contrast-media-induced acute kidney injury: Pathophysiology and prophylactic strategies. ISRN Radiol.

[16] Andreucci M., Solomon R., Tasanarong A. (2014). Side effects of radiographic contrast media: Pathogenesis, risk factors, and prevention. Biomed. Res. Int..

[17] Quintavalle C., Brenca M., De Micco F., Fiore D., Romano S., Romano M.F., Apone F., Bianco A., Zabatta M.A., Troncone G. (2011). In vivo and in vitro assessment of pathways involved in contrast media-induced renal cells apoptosis. Cell Death Dis..

[18] Yokomaku Y., Sugimoto T., Kume S., Araki S., Isshiki K., Chin-Kanasaki M., Sakaguchi M., Nitta N., Haneda M., Koya D. (2008). Asialoerythropoietin prevents contrast-induced nephropathy. J. Am. Soc. Nephrol..

[19] Sadat U., Usman A., Boyle J.R., Hayes P.D., Solomon R.J. (2015). Contrast Medium-Induced Acute Kidney Injury. Cardiorenal Med..

[20] Briguori C., Donnarumma E., Quintavalle C., Fiore D., Condorelli G. (2015). Contrast-induced acute kidney injury: Potential new strategies. Curr. Opin. Nephrol. Hypertens..

[21] Lee H.C., Sheu S.H., Liu I.H., Lee C.C., Hsieh C.C., Yen H.W., Lai W.T., Chang J.G. (2012). Impact of short-duration administration of *N*-acetylcysteine, probucol and ascorbic acid on contrast-induced cytotoxicity. J. Nephrol..

[22] Dodd S., Dean O., Copolov D.L., Malhi G.S., Berk M. (2008). *N*-acetylcysteine for antioxidant therapy: Pharmacology and clinical utility. Expert Opin. Biol. Ther..

[23] Sun Y., Pu L.Y., Lu L., Wang X.H., Zhang F., Rao J.H. (2014). *N*-acetylcysteine attenuates reactive-oxygen-species-mediated endoplasmic reticulum stress during liver ischemia-reperfusion injury. World J. Gastroenterol..

[24] Ratliff B.B., Abdulmahdi W., Pawar R., Wolin M.S. (2016). Oxidant mechanisms in renal injury and disease. Antioxid. Redox Signal.

[25] Romano G., Briguori C., Quintavalle C., Zanca C., Rivera N.V., Colombo A., Condorelli G. (2008). Contrast agents and renal cell apoptosis. Eur. Heart J..

[26] Park J.Y., Lee D., Jang H.J., Jang D.S., Kwon H.C., Kim K.H., Kim S.N., Hwang G.S., Kang K.S., Eom D.W. (2015). Protective effect of artemisia asiatica extract and its active compound eupatilin against cisplatin-induced renal damage. Evid. Based Complement. Alternat. Med..

[27] Jeong E.K., Jang H.J., Kim S.S., Oh M.Y., Lee D.H., Eom D.W., Kang K.S., Kwan H.C., Ham J.Y., Park C.S. (2015). Protective effect of eupatilin against renal ischemia-reperfusion injury in mice. Transpl. Proc..

[28] He X.Y., Li L.W., Tan H., Chen J.Y., Zhou Y.L. (2016). Atorvastatin attenuates contrast-induced nephropathy by modulating inflammatory responses through the regulation of JNK/p38/Hsp27 expression. J. Pharmacol. Sci..

[29] Shen J., Wang L., Jiang N., Mou S., Zhang M., Gu L., Shao X., Wang Q., Qi C., Li S. (2016). NLRP3 inflammasome mediates contrast media-induced acute kidney injury by regulating cell apoptosis. Sci. Rep..

[30] Seeliger E., Sendeski M., Rihal C.S., Persson P.B. (2012). Contrast-induced kidney injury: Mechanisms, risk factors, and prevention. Eur. Heart J..

[31] Hizoh I., Haller C. (2002). Radiocontrast-induced renal tubular cell apoptosis: Hypertonic versus oxidative stress. Investig. Radiol..

[32] Hizoh I., Strater J., Schick C.S., Kubler W., Haller C. (1998). Radiocontrast-induced DNA fragmentation of renal tubular cells in vitro: Role of hypertonicity. Nephrol. Dial Transpl..

[33] Guo S.X., Fang Q., You C.G., Jin Y.Y., Wang X.G., Hu X.L., Han C.M. (2015). Effects of hydrogen-rich saline on early acute kidney injury in severely burned rats by suppressing oxidative stress induced apoptosis and inflammation. J. Transl. Med..

[34] Dabaghi-Barbosa P., Mariante Rocha A., Franco da Cruz Lima A., Heleno de Oliveira B., Benigna Martinelli de Oliveira M., Gunilla Skare Carnieri E., Cadena S.M., Eliane Merlin Rocha M. (2005). Hispidulin: Antioxidant properties and effect on mitochondrial energy metabolism. Free Radic. Res..

[35] Lai Z.R., Ho Y.L., Huang S.C., Huang T.H., Lai S.C., Tsai J.C., Wang C.Y., Huang G.J., Chang Y.S. (2011). Antioxidant, anti-inflammatory and antiproliferative activities of *Kalanchoe gracilis* (L.) DC stem. Am. J. Chin. Med..

[36] Park Y., Lee S., Woo Y., Lim Y. (2009). Relationships between Structure and Anti-oxidative Effects of Hydroxyflavones. Bull. Korean Chem. Soc..

[37] Small D.M., Morais C., Coombes J.S., Bennett N.C., Johnson D.W., Gobe G.C. (2014). Oxidative stress-induced alterations in PPAR-gamma and associated mitochondrial destabilization contribute to kidney cell apoptosis. Am. J. Physiol.-Renal Physiol..

[38] Kiss-Toth E., Roszer T. (2008). PPAR gamma in kidney physiology and pathophysiology. PPAR Res..

[39] Panchapakesan U., Pollock C.A., Chen X.M. (2004). The effect of high glucose and PPAR-gamma agonists on PPAR-gamma expression and function in HK-2 cells. Am. J. Physiol.-Renal Physiol..

[40] Wen L.L., Lin C.Y., Chou H.C., Chang C.C., Lo H.Y., Juan S.H. (2016). Perfluorooctanesulfonate Mediates Renal Tubular Cell Apoptosis through PPARgamma Inactivation. PLoS ONE.

[41] Hong W., Hu S., Zou J., Xiao J., Zhang X., Fu C., Feng X., Ye Z. (2015). Peroxisome proliferator-activated receptor gamma prevents the production of NOD-like receptor family, pyrin domain containing 3 inflammasome and interleukin 1beta in HK-2 renal tubular epithelial cells stimulated by monosodium urate crystals. Mol. Med. Rep..

[42] Lepenies J., Hewison M., Stewart P.M., Quinkler M. (2010). Renal PPAR gamma mRNA expression increases with impairment of renal function in patients with chronic kidney disease. Nephrology.

[43] Tarawneh A., Leon F., Pettaway S., Elokely K.M., Klein M.L., Lambert J., Mansoor A., Cutler S.J. (2015). Flavonoids from Perovskia atriplicifolia and their in vitro displacement of the respective radioligands for human opioid and cannabinoid receptors. J. Nat. Prod..

[44] Nakasugi T., Nakashima M., Komai K. (2000). Antimutagens in gaiyou (Artemisia argyi levl. et vant.). J. Agric. Food Chem..

[45] He F., Aisa H.A., Shakhidoyatov K.M. (2012). Flavones from Artemisia rupestris. Chem. Nat. Compd..

[46] Yu H., Chen J., Xu X., Li Y., Zhao H., Fang Y., Li X., Zhou W., Wang W., Wang Y. (2012). A systematic prediction of multiple drug-target interactions from chemical, genomic, and pharmacological data. PLoS ONE.

[47] Liu Y., Mabry T.J. (1981). Flavonoids from Artemisia-Frigida. Phytochemistry.

[48] Elansari M.A., Barron D., Abdalla M.F., Saleh N.A.M., Lequere J.L. (1991). Flavonoid constituents of stachys-aegyptiaca. Phytochemistry.

[49] Martinez V., Barbera O., Sanchezparareda J., Marco J.A. (1987). Phenolic and acetylenic metabolites from Artemisia-Assoana. Phytochemistry.

[50] Hanamura S., Hanaya K., Shoji M., Sugai T. (2016). Synthesis of acacetin and resveratrol 3,5-di-O-beta-glucopyranoside using lipase-catalyzed regioselective deacetylation of polyphenol glycoside peracetates as the key step. J. Mol. Catal. B Enzym..

[51] Ibrahim A.R., Galal A.M., Ahmed M.S., Mossa G.S. (2003). *O*-demethylation and sulfation of 7-methoxylated flavanones by Cunninghamella elegans. Chem. Pharm. Bull. (Tokyo).

[52] Ribeiro D., Freitas M., Tome S.M., Silva A.M., Porto G., Fernandes E. (2013). Modulation of human neutrophils’ oxidative burst by flavonoids. Eur. J. Med. Chem..

[53] Lee S.J., Kim H.M., Lee S., Kim H.Y., Um B.H., Ahn Y.H. (2006). Apicin, a new flavonoid from Artemisia apiacea. Bull. Korean Chem. Soc..

[54] Shu R.G., Hu H.W., Zhang P.Z., Ge F. (2012). Triterpenes and flavonoids from Mosla chinensis. Chem. Nat. Compd..

[55] Turkkan B., Ozyurek M., Bener M., Guclu K., Apak R. (2012). Synthesis, characterization and antioxidant capacity of naringenin-oxime. Spectrochim. Acta A Mol. Biomol. Spectrosc..

[56] Shi Z.H., Li N.G., Wang Z.J., Tang Y.P., Dong Z.X., Zhang W., Zhang P.X., Gu T., Wu W.Y., Yang J.P. (2015). Synthesis and biological evaluation of methylated scutellarein analogs based on metabolic mechanism of scutellarin in vivo. Eur. J. Med. Chem..

[57] Amrutha K., Nanjan P., Shaji S.K., Sunilkumar D., Subhalakshmi K., Rajakrishna L., Banerji A. (2014). Discovery of lesser known flavones as inhibitors of NF-kappa B signaling in MDA-MB-231 breast cancer cells-A SAR study. Bioorg. Med. Chem. Lett..

[58] Nagao T., Abe F., Kinjo J., Okabe H. (2002). Antiproliferative constituents in plants 10. Flavones from the leaves of Lantana montevidensis BRIQ. and consideration of structure-activity relationship. Biol. Pharm. Bull..

[59] Teles Y.C.F., Horta C.C.R., Agra M.D., Siheri W., Boyd M., Igoli J.O., Gray A.I., de Souza M.D.V. (2015). New sulphated flavonoids from *Wissadula periplocifolia* (L.) C. Presl (Malvaceae). Molecules.

[60] Kyriakou E., Primikyri A., Charisiadis P., Katsoura M., Gerothanassis I.P., Stamatis H., Tzakos A.G. (2012). Unexpected enzyme-catalyzed regioselective acylation of flavonoid aglycones and rapid product screening. Org. Biomol. Chem..

[61] Li H.Z., Zhang Y.Q., Liu Q., Sun C.L., Li J., Yang P., Wang X. (2016). Preparative separation of phenolic compounds from chimonanthus praecox flowers by high-speed counter-current chromatography using a stepwise elution mode. Molecules.

[62] Lee D., Choi Y.O., Kim K.H., Chin Y.-W., Namgung H., Yamabe N., Jung K. (2016). Protective effect of α-mangostin against iodixanol-induced apoptotic damage in LLC-PK1 cells. Bioorg. Med. Chem. Lett..

[63] Lee H.L., Kang K.S. (2017). Protective effect of ginsenoside Rh3 against anticancer drug-induced apoptosis in LLC-PK1 kidney cells. J. Ginseng. Res..

[64] Jeon J.H., Kim D.K., Shin Y., Kim H.Y., Song B., Lee E.Y., Kim J.K., You H.J., Cheong H., Shin D.H. (2016). Migration and invasion of drug-resistant lung adenocarcinoma cells are dependent on mitochondrial activity. Exp. Mol. Med..

[65] Xu X., Zhang W., Huang C., Li Y., Yu H., Wang Y., Duan J., Ling Y. (2012). A novel chemometric method for the prediction of human oral bioavailability. Int. J. Mol. Sci..

[66] Lipinski C.A., Lombardo F., Dominy B.W., Feeney P.J. (2001). Experimental and computational approaches to estimate solubility and permeability in drug discovery and development settings. Adv. Drug Deliv. Rev..

[67] Yamanishi Y., Kotera M., Kanehisa M., Goto S. (2010). Drug-target interaction prediction from chemical, genomic and pharmacological data in an integrated framework. Bioinformatics.

