# Supplementary material for: Protective Effect of Artemisia argyi and Its Flavonoid Constituents against Contrast-Induced Cytotoxicity by Iodixanol in LLC-PK1 Cells
Source: Int J Mol Sci. 2018 May 7;19(5):1387. doi: 10.3390/ijms19051387 (PMC5983776; doi:10.3390/ijms19051387)

Supplementary Materials

Protective Effect of *Artemisia argyi* and Its Flavonoid Constituents against Contrast-Induced Cytotoxicity by Iodixanol in LLC-PK1 Cells

Dahae Lee [1,†], Chang-Eop Kim [2,†], Sa-Yoon Park [2], Kem Ok Kim [3], Nguyen Tuan Hiep [3], Dongho Lee [3], Hyuk-Jai Jang [4], Jae Wook Lee [5,*] and Ki Sung Kang [2,*]

[1] School of Pharmacy, Sungkyunkwan University, Suwon 440-746, Korea; pjsldh@naver.com
[2] College of Korean Medicine, Gachon University, Seongnam 13120, Korea; eopchang@gachon.ac.kr (C.E.K.); psy9228@gmail.com (S.Y.P.)
[3] Department of Biosystems and Biotechnology, College of Life Science and Biotechnology, Korea University, Seoul 02841, Korea; kikeko520@gmail.com (K.O.K.); nguyentuanhiep.2710@gmail.com (N.T.H.); dongholee@korea.ac.kr (D.L.)
[4] Department of Surgery, University of Ulsan College of Medicine, Seoul 05505, Korea; jhj@gnah.co.kr
[5] Natural Constituent Research Center, Korea Institute of Science and Technology, Gangnung 210-340, Korea

---

S1. ^{1}H NMR spectroscopic data of compounds **1–19** in DMSO-d_6.

3′-*O*-Methyl-eupatorin (**1**): ^{1}H-NMR *δ* 12.91 (1H, *s*, 5-OH), 7.75 (1H, *dd*, *J* = 8.5, 2 Hz, H-6′), 7.61 (1H, *d*, H-2′), 7.15 (1H, *d*, *J* = 8.5 Hz, H-5′), 7.06 (1H, *s*, H-3), 6.99 (1H, *s*, H-8), 3.95 (3H, *s*, OMe-7), 3.90 (3H, *s*, OMe-3′), 3.87 (3H, *s*, OMe-4′), 3.74 (3H, *s*, OMe-6).

Artemetin (**2**): ^{1}H-NMR *δ* 12.60 (1H, *s*, 5-OH), 7.75 (1H, *d*, *J* = 8 Hz, H-6′), 7.67 (1H, *s*, H-2′), 7.18 (1H, *d*, *J* = 8 Hz, H-5′), 6.96 (1H, *s*, H-8), 3.94 (3H, *s*, OMe-7), 3.87 (6H, *s*, OMe-3′, 4′), 3.83 (3H, *s*, OMe-3), 3.74 (3H, *s*, OMe-6).

Chrysosplenetin (**3**): ^{1}H-NMR *δ* 12.64 (1H, *s*, 5-OH), 7.68 (1H, *d*, *J* = 2 Hz, H-2′), 7.65 (1H, *dd*, *J* = 8.5, 2 Hz, H-6′), 6.98 (1H, *d*, *J* = 8.5 Hz, H-5′), 6.94 (1H, *s*, H-8), 3.93 (3H, *s*, OMe-7), 3.87 (6H, *s*, OMe-3′), 3.82 (3H, *s*, OMe-3), 3.73 (3H, *s*, OMe-6).

Eupatilin (**4**): ^{1}H-NMR *δ* 13.04 (1H, *s*, 5-OH), 10.66 (1H, *s*, 7-OH), 7.68 (1H, *dd*, *J* = 8.5, 1.5 Hz, H-6′), 7.56 (1H, *d*, *J* = 1.5 Hz, H-2′), 7.13 (1H, *d*, *J* = 8.5 Hz, H-5′), 6.95 (1H, *s*, H-3), 6.63 (1H, *s*, H-8), 3.89 (6H, *s*, OMe-3′), 3.86 (6H, *s*, OMe-4′), 3.77 (3H, *s*, OMe-6).

Bonanzin (**5**): ^{1}H-NMR *δ* 12.70 (1H, *s*, 5-OH), 10.70 (1H, *s*, 7-OH), 7.67 (1H, *dd*, *J* = 8.5, 2 Hz, H-6′), 7.61 (1H, *d*, *J* = 2 Hz, H-2′), 7.15 (1H, *d*, *J* = 8.5 Hz, H-5′), 6.58 (1H, *s*, H-8), 3.84 (6H, *s*, OMe-4′), 3.83 (6H, *s*, OMe-3′), 3.79 (3H, *s*, OMe-6), 3.74 (3H, *s*, OMe-3).

Eupafolin (6): ^{1}H-NMR *δ* 13.09 (1H, *s*, 5-OH), 7.41 (1H, *dd*, *J* = 8.5, 2.5 Hz, H-2′), 7.39 (1H, *d*, *J* = 2.5 Hz, H-6′), 6.88 (1H, *d*, *J* = 8.5 Hz, H-3′), 6.67 (1H, *s*, H-3), 6.54 (1H, *s*, H-8), 3.74 (3H, *s*, OMe-6).

3,6,3′-Trimethoxy-5,7,4′-trihydroxyflavone (7): ^{1}H-NMR *δ* 12.76 (1H, *s*, 5-OH), 7.63 (1H, *d*, *J* = 1 Hz, H-2′), 7.57 (1H, *dd*, *J* = 8.5, 1 Hz, H-6′), 6.96 (1H, *d*, *J* = 8.5 Hz, H-5′), 6.58 (1H, *s*, H-8), 3.85 (3H, *s*, OMe-3′), 3.79 (3H, *s*, OMe-3), 3.74 (3H, *s*, OMe-6).

Acacetin (8): ^{1}H-NMR *δ* 12.93 (1H, *s*, 5-OH), 10.87 (1H, *s*, 7-OH), 8.06 (2H, *d*, *J* = 8.5 Hz, H-2′, 6′), 7.13 (2H, *d*, *J* = 8.5 Hz, H-3′, 5′), 6.89 (1H, *s*, H-3), 6.51 (1H, *s*, H-8), 6.21 (1H, *s*, H-6), 3.86 (3H, *s*, OMe-4′).

Homoeriodictyol (9): ^{1}H-NMR *δ* 12.15 (1H, *s*, 5-OH), 9.15 (1H, *s*, 4′-OH), 7.08 (1H, *dd*, *J* = 2 Hz, H-2′), 6.90 (1H, *d*, *J* = 8, 2 Hz, H-6′), 6.79 (1H, *d*, *J* = 8 Hz, H-5′), 5.89 (2H, *m*, H-6, 8), 5.43 (1H, *dd*, *J* = 13, 2.5 Hz, H-2), 3.32 (1H, *m*, H-3), 2.68 (1H, *dd*, *J* = 17, 2.5 Hz, H-3), 3.78 (3H, *s*, OMe-3′).

Jaceosidin (10): ^{1}H-NMR *δ* 13.10 (1H, *s*, 5-OH), 10.70 (1H, *s*, 7-OH), 7.58 (1H, *m*, H-6′), 7.56 (1H, *m*, H-2′), 6.95 (1H, *d*, *J* = 8.5 Hz, H-5′), 6.92 (1H, *s*, H-3), 6.63 (1H, *s*, H-8), 3.90 (3H, *s*, OMe-3′), 3.76 (3H, *s*, OMe-6).

3′,4′-Dimethoxyluteolin (11): ^{1}H-NMR *δ* 12.94 (1H, *s*, 5-OH), 10.88 (1H, br *s*, 7-OH), 7.70 (1H, *dd*, *J* = 8.5, 2 Hz, H-6′), 7.57 (1H, *d*, *J* = 2 Hz, H-2′), 7.14 (1H, *d*, *J* = 8.5 Hz, H-5′), 6.99 (1H, *s*, H-3), 6.54 (1H, *d*, *J* = 2 Hz, H-8), 6.21 (1H, *d*, *J* = 2 Hz, H-6), 3.88 (3H, *s*, OMe-3′), 3.86 (3H, *s*, OMe-4′).

3′-Methoxyapigenin (12): ^{1}H-NMR *δ* 12.96 (1H, *s*, 5-OH), 7.56 (1H, *dd*, *J* = 9, 2 Hz, H-6′), 7.54 (1H, *m*, H-2′), 6.93 (1H, *d*, *J* = 9 Hz, H-5′), 6.88 (1H, *s*, H-3), 6.47 (1H, *d*, *J* = 2 Hz, H-8), 6.16 (1H, *d*, *J* = 1.5 Hz, H-6), 3.88 (3H, *s*, OMe-3′).

Naringenin (13): ^{1}H-NMR *δ* 12.15 (1H, *s*, 5-OH), 9.61 (1H, *s*, 4′-OH), 7.31 (2H, *d*, *J* = 8.5 Hz, H-2′, 6′), 6.79 (2H, *d*, *J* = 8.5 Hz, H-3′, 5′), 5.88 (2H, *m*, H-6, H-8), 5.44 (1H, *dd*, *J* = 13, 3 Hz, H-2), 3.29 (1H, *dd*, *J* = 17, 12.5 Hz, H-3), 2.68 (1H, *dd*, *J* = 17, 3 Hz, H-3).

Hispidulin (14): ^{1}H-NMR *δ* 13.09 (1H, *s*, 5-OH), 7.94 (2H, *d*, *J* = 8.5 Hz, H-2′, 6′), 6.94 (2H, *d*, *J* = 9 Hz, H-3′, 5′), 6.79 (1H, *s*, H-3), 6.60 (1H, *s*, H-8), 3.75 (3H, *s*, OMe-6).

5,7,3′,4′-Tetrahydroxyflavanone (15): ^{1}H-NMR *δ* 12.15 (1H, *s*, 5-OH), 10.80 (1H, *s*, 7-OH), 9.10 (1H, s, 3'-OH), 9.04 (1H, s, 4'-OH), 6.87 (1H, *s*, H-5′), 6.74 (2H, *s*, H-2′, 6′), 5.88 (2H, *s*, H-6, 8), 5.39 (1H, *dd*, *J* = 12.5, 3 Hz, H-2), 3.22 (1H, *dd*, *J* = 17, 12.5 Hz, H-3), 2.69 (1H, *dd*, *J* = 17, 3 Hz, H-3).

3,4′,5,7-Tetrahydroxyflavone (16): ^{1}H-NMR *δ* 12.49 (1H, *s*, 5-OH), 10.82 (1H, *s*, 7-OH), 9.42 (1H, *s*, 4′-OH), 8.05 (2H, *d*, *J* = 8.5 Hz, H-2′, 6′), 6.94 (2H, *d*, *J* = 9 Hz, H-3′, 5′), 6.44 (1H, *d*, *J* = 1 Hz, H-8), 6.19 (1H, *d*, *J* = 1 Hz, H-6).

5,7,4′-Trihydroxyflavone (17): ^{1}H-NMR *δ* 12.97 (1H, *s*, 5-OH), 10.86 (1H, *s*, 7-OH), 7.94 (2H, *d*, *J* = 8.5 Hz, H-2′, H-6′), 6.94 (1H, *d*, *J* = 8.5 Hz, H-3′, H-5′), 6.80 (1H, *s*, H-3), 6.49 (1H, *d*, *J* = 2 Hz, H-8), 6.20 (1H, *d*, *J* = 2 Hz, H-6).

2,3-Dihydroisorhamnetin (18): ^{1}H-NMR *δ* 11.93 (1H, *s*, 5-OH), 9.14 (1H, *s*, 4′-OH), 7.11 (1H, *d*, *J* = 1.5 Hz, H-2′), 6.91 (1H, *dd*, *J* = 8.5, 1.5 Hz, H-6′), 6.79 (1H, *d*, *J* = 8.5 Hz, H-5′), 5.92 (1H, *d*, *J* = 2 Hz, H-6), 5.87 (1H, *d*, *J* = 1.5 Hz, H-8), 5.77 (1H, *d*, 3-OH), 5.05 (1H, *d*, *J* = 11.5 Hz, H-2), 4.66 (1H, *dd*, *J* = 11.5, 6 Hz, H-3), 3.78 (3H, *s*, OMe-3′).

Apicin (19): ^{1}H-NMR *δ* 13.07 (1H, *s*, 5-OH), 7.46 (1H, *s*, H-6′), 7.12 (1H, *s*, H-2′), 6.98 (1H, *s*, H-8), 6.57 (1H, *s*, H-4′), 3.94 (3H, *s*, OMe-7), 3.82 (3H, *s*, OMe-5′), 3.73 (3H, *s*, OMe-6).

S2. Full-length gels for Figure 3.

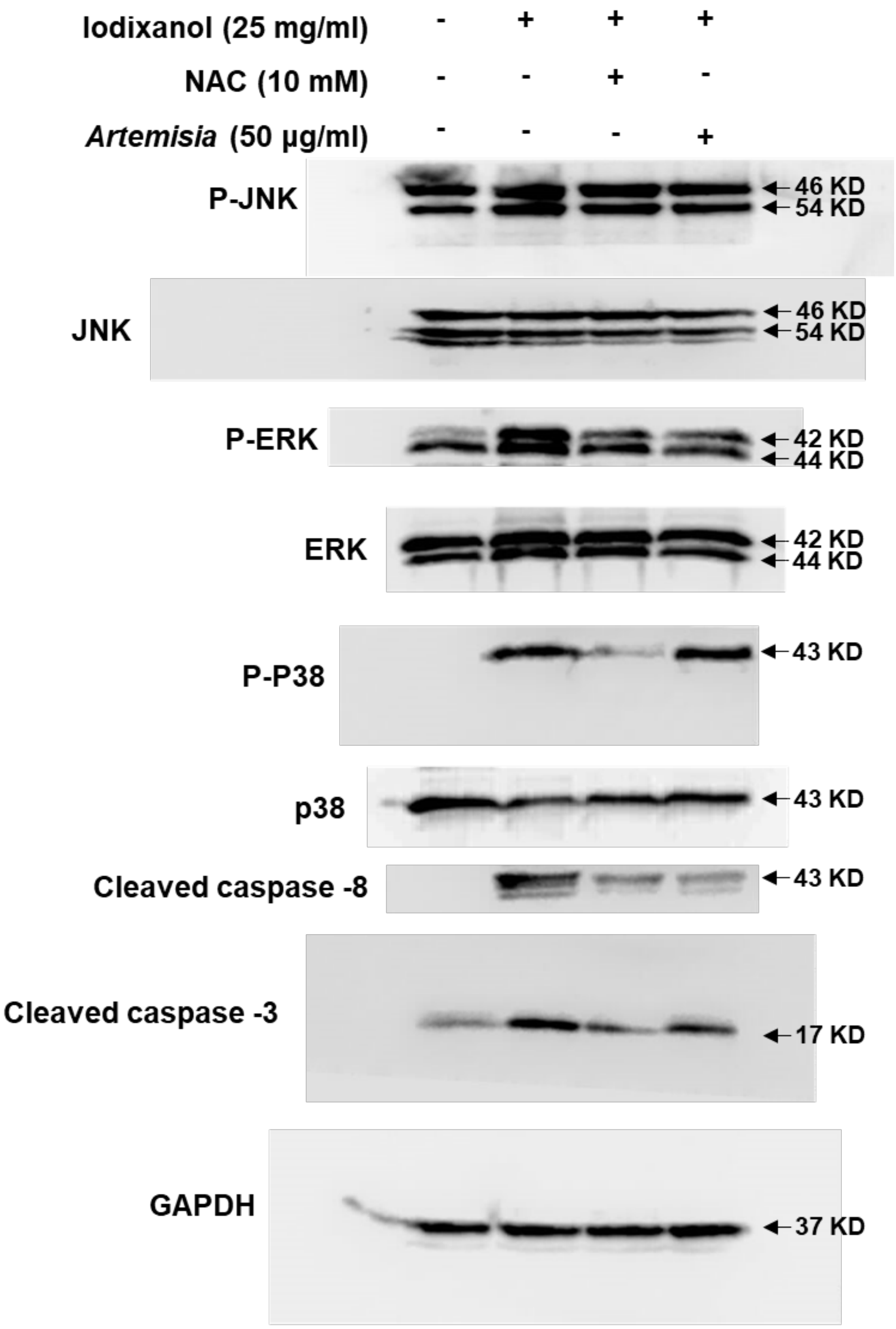

S3. Full-length gels for Figure 6.

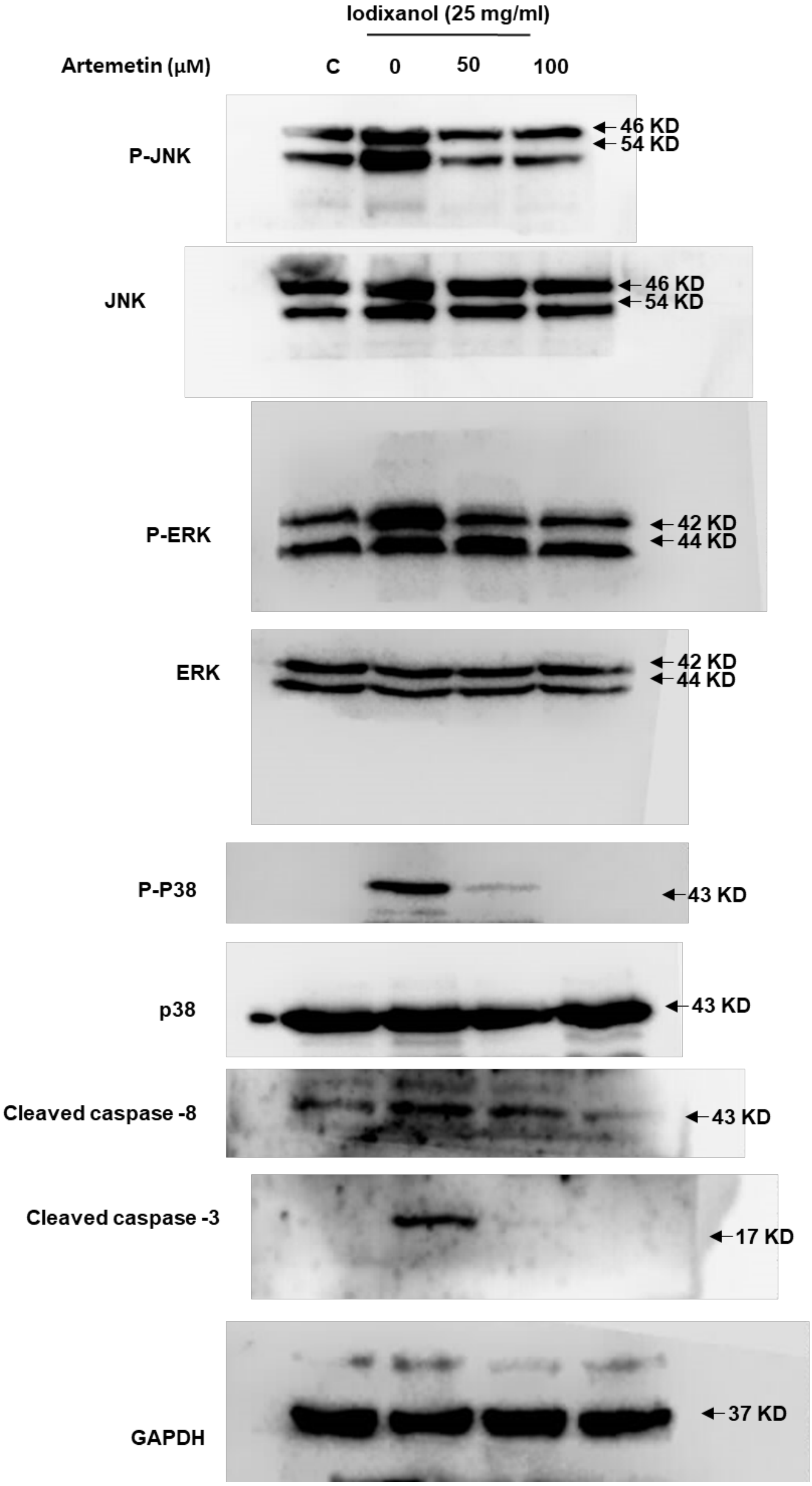

Supplement: Supplementary file 1 [file ijms-19-01387-s001.pdf]
